# A narrative review of Phase III and IV clinical trials for the pharmacological treatment of Tourette’s syndrome in children, adults, and older adults

**DOI:** 10.1097/MD.0000000000042760

**Published:** 2025-06-06

**Authors:** Mohammed S. Alharthi

**Affiliations:** a Department of Clinical Pharmacy, College of Pharmacy, Taif University, Taif, Saudi Arabia.

**Keywords:** clinical trials, neurodevelopmental disorder, pharmacological treatments, Phase III, Phase IV, tics, Tourette syndrome

## Abstract

Tourette syndrome (TS) is a neurodevelopmental disorder marked by involuntary movements and vocalizations (tics), often accompanied by cognitive and conduct challenges. Despite existing treatments, many remain ineffective, prompting increased focus on Phase III and IV clinical trials for improved pharmacological options. These trials aim to refine symptom management and enhance the quality of life for individuals with TS. This review aims to identify the pharmacological treatments trialed in completed Phase III and IV clinical trials for managing TS in children, adults, and older adults. Phase III and IV clinical trials registered at ClinicalTrials.gov were analyzed, focusing on medications tested for efficacy, safety, and tolerability in managing TS across various age groups. As of February 2025, 15 relevant Phase III and IV trials were reviewed. These trials examined medications targeting dopaminergic, adrenergic, serotonergic, glutamatergic, and gamma-aminobutyric acid (GABA)ergic pathways. Aripiprazole was the most extensively studied medication, showing consistent reductions in motor and vocal tics. Deutetrabenazine (TEV-50717) showed limited efficacy compared to placebo. Other medications, including Clonidine, Guanfacine, Levetiracetam, Ondansetron, Topiramate, D-serine, and Riluzole, demonstrated varying levels of effectiveness, though some require further validation. Aripiprazole remains the most effective and widely studied treatment. Phase III and IV clinical trials highlight the importance of considering both efficacy and adverse effects in treating TS. Aripiprazole stands out as the most effective therapy, though further research is needed for other medications. Continued advancements in pharmacological interventions offer promise for improving the management of TS and enhancing the quality of life for affected individuals.

## 1. Introduction

Tourette syndrome (TS) is a rare neurological disorder characterized by repetitive, involuntary movements and vocalizations, commonly known as tics.^[1]^ First described in the 19th century by French neurologist Dr Georges Gilles de la Tourette, this condition, though uncommon, profoundly impacts populations.^[2]^ The hallmark of TS is the occurrence of sudden, rapid, and recurrent tics, which can range from mild to severe, involving both motor and vocal expressions. Motor tics can include physical movements such as blinking, head jerking, and shoulder shrugging, while vocal tics may involve throat clearing, grunting, or socially inappropriate phrases (coprolalia).^[3]^ Typically, TS manifests in childhood, with symptoms often peaking between the ages of 5 and 10 years.^[4]^ The syndrome’s cause is multifactorial, involving genetic predispositions and environmental factors. A family history of tic disorders increases the likelihood of developing TS.^[5]^ Additionally, abnormalities in specific brain regions, neurotransmitter functioning, and nervous system development contribute to the expression of tics.^[6]^ Beyond the physical symptoms, individuals with TS often face significant social challenges, including misunderstandings and disruptions to daily life.^[7]^ Raising awareness about the condition is essential in fostering inclusive societies that recognize and address the difficulties faced by those affected.^[8]^ While the severity and frequency of tics can fluctuate over time, many individuals experience symptom improvement during adolescence or adulthood.^[9]^ However, challenges may persist throughout adulthood, impacting social relationships, academic or professional performance, and overall well-being.^[10]^ Treatment of TS requires a comprehensive approach, combining behavioral therapy, psycho-education, and in some instances, medication.^[11]^ Although there is no known cure for TS, treatment strategies focus on symptom management and improving the quality of life.^[12]^ Efforts to reduce stigma and enhance public understanding have led to earlier identification and more timely interventions.^[13]^ Public awareness also promotes a more inclusive environment, helping individuals with TS confidently manage their condition.^[14]^Recent research continues to deepen our understanding of the disorder’s underlying mechanisms, paving the way for advancements in therapeutic approaches. This narrative review aims to explore and analyze Phase III and IV pharmacological treatments for TS, focusing on their efficacy, safety, and tolerability in children, adults, and older adults. The goal is to provide a comprehensive overview of current and potential therapeutic strategies for managing this complex disorder.

## 2. Methods

### 2.1. Data source and search strategy

ClinicalTrials.gov served as the primary data source for this review. It is a comprehensive public registry cataloguing clinical trials across 221 countries, encompassing medical studies involving human volunteers and interventional studies that report results publicly. The search for relevant studies was conducted from the database’s inception until February 11, 2025. This search retrieved data from 60 relevant studies concerning TS, using keywords such as “Tourette syndrome,” “tic disorders,” and “interventional studies.”

Inclusion criteria were defined as follows:

- Primary focus on TS and tic-related disorders.
- Studies in Phase III and IV.
- Inclusion of children, adults, and older adults.
- Availability of study results on ClinicalTrials.gov.

**Figure 1.**
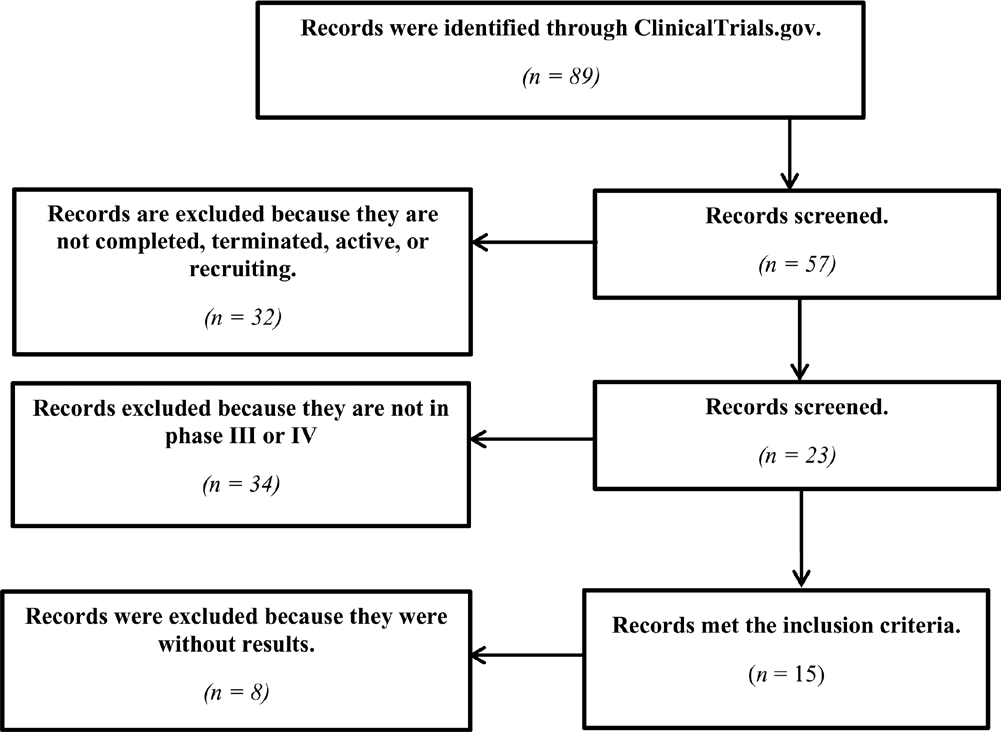
Summary of the review process. This figure illustrates the review’s step-by-step process, covering records identification, screening, and applying inclusion criteria to clinical trials. It summarizes study exclusions based on predefined criteria, including phase designation, population age range, and study completion status.

The review process is summarized in Figure 1.

### 2.2. Data collection and coding

Data collection and coding followed a systematic approach. Key parameters documented for each study included the study title, study design, interventions, age group, summary, and primary outcome measures. The data collected included information on treatment types (e.g., Aripiprazole, Deutetrabenazine, Topiramate), mechanisms of action, dosage, treatment duration, study results, and associated side effects. All information was manually coded to ensure accuracy and consistency in categorization for analysis.

### 2.3. Study design selection

Studies were selected based on predetermined criteria. This review focused on Phase III and IV trials investigating the efficacy and safety of pharmacological treatments for TS. Phase I and II studies were excluded to maintain the focus on treatments with more established clinical data. The selection process ensured a thorough analysis of the’ efficacy and side effect profiles of pharmacological interventions.

## 3. Results

### 3.1. Overview

This study examined 15 studies that fulfilled the inclusion criteria after excluding others. Phase III and IV clinical trials explored pharmacological treatments for TS, targeting dopaminergic, adrenergic, serotonergic, glutamatergic, and gamma-aminobutyric acid (GABA)ergic pathways to reduce tics. Phase III trials assessed Aripiprazole and Deutetrabenazine, with Aripiprazole consistently demonstrating tic reduction, while Deutetrabenazine showed no significant benefit over placebo. Phase IV trials examined the long-term efficacy and safety of Levetiracetam, Clonidine, Ondansetron, and Guanfacine. Clonidine and Guanfacine were effective for vocal tics and attention-deficit/hyperactivity disorder (ADHD) symptoms but caused sedation, while Levetiracetam showed inconsistent results. Ondansetron had variable effects with frequent gastrointestinal side effects. Topiramate, D-serine, and Riluzole showed potential benefits, though further validation is needed. Aripiprazole remains the most extensively studied and consistently effective treatment.

### 3.2. Comparison of Tourette syndrome treatments

To reduce tic severity, pharmacological treatments for TS target dopaminergic, adrenergic, serotonergic, glutamatergic, and GABAergic pathways. Table 1 provides a comparative analysis of these treatments. Aripiprazole, the most extensively studied drug, significantly reduces both motor and vocal tics with a favorable safety profile. Deutetrabenazine, a vesicular monoamine transporter 2 inhibitor, showed limited efficacy. Clonidine and Guanfacine, both alpha-2 adrenergic agonists, are effective for vocal tics and ADHD symptoms but commonly cause sedation. Levetiracetam has inconsistent effects, while Ondansetron, a serotonin modulator, provides variable tic reduction but frequently causes gastrointestinal side effects. Topiramate and glutamatergic modulators (D-serine and Riluzole) show potential but require further research.

**Table 1:** Comparative pharmacological treatments for Tourette syndrome: mechanisms, efficacy, and outcomes.

Medication	Mechanism of action	Effects on tics	Dosage and treatment duration	Treatment outcomes
Levetiracetam	Binds to synaptic vesicle protein 2A (SV2A), modulating neurotransmitter release.^[1]^	Mixed results: some trials show a modest reduction in motor tics but no significant effect on vocal tics.	250–1000 mg/day for 8–12 weeks.	Some trials report tic reduction, particularly for motor tics, but overall efficacy remains inconsistent.
Clonidine	Alpha-2 adrenergic agonist reduces noradrenergic activity, stabilizing neurotransmitter fluctuations.^[2]^	It is more effective for vocal tics and tic-related impulsivity, particularly in ADHD patients.	0.05–0.3 mg/day for 6 weeks.	Modest efficacy: beneficial for patients with coexisting ADHD symptoms but limited for tic suppression alone.
Ondansetron	5-HT3 receptor antagonists modulate serotonin pathways in neural circuits.^[3]^	Though efficacy is inconsistent, there is some improvement in both motor and vocal tics.	4–16 mg/day for 8–12 weeks.	Varied effectiveness: some reduction in tic severity, but gastrointestinal side effects are common.
Aripiprazole	Partial dopamine D2/D3 receptor agonist and serotonin modulator, stabilizing dopamine levels.^[4]^	It significantly reduces motor and vocal tics, and higher doses are more effective.	1–20 mg/day (daily) or 52.5–110 mg/week for 6–24 weeks.	Consistently effective in reducing tic severity, with a strong balance of efficacy and tolerability.
Deutetrabenazine (TEV-50717)	Vesicular monoamine transporter 2 inhibitors reduce dopamine release to control excessive neurotransmission.	It did not show significant improvement over the placebo; it had a limited effect on motor and vocal tics.	6–48 mg/day for 12 weeks.	There is no significant advantage over placebo; common side effects include fatigue and nausea.
Topiramate	Modulates GABAergic and glutamatergic neurotransmission to reduce excitatory signaling.^[5]^	Effective for motor tics with some benefit for vocal tics; limited by cognitive side effects.	25–200 mg/day for 8–12 weeks.	Demonstrates tic reduction, but cognitive slowing and drowsiness limit long-term use.
Guanfacine	Alpha-2 is an adrenergic agonist similar to clonidine, reducing sympathetic activity.^[6]^	Reduces tics in children with ADHD; more effective for mild cases but causes sedation.	1–4 mg/day for 8–12 weeks.	It is effective for tics in ADHD patients but has high rates of sedation and fatigue.
D-serine & Riluzole	Modulates glutamatergic neurotransmission, influencing tic-related neural pathways.^[7]^	Some reduction in tics, particularly in patients with OCD symptoms; further research is needed.	D-serine: 30 mg/kg/day; Riluzole: 50–200 mg/day for 8 weeks.	Preliminary evidence suggests potential efficacy, but more extensive trials are required.

### 3.3. Clinical trials on pharmacological treatments for Tourette syndrome

Clinical trials investigating pharmacological treatments for TS evaluate their efficacy, safety, and tolerability across different study phases. Table 2 summarizes key trials, categorizing them based on their design, interventions, and study locations. Aripiprazole is the most extensively studied drug, consistently demonstrating tic reduction across multiple dosing regimens. Deutetrabenazine and topiramate also show promise, with topiramate potentially improving attention and obsessive–compulsive symptoms. Ondansetron, guanfacine, and glutamatergic modulators (riluzole and D-serine) have been explored, but their efficacy requires further research. Levetiracetam is a potential alternative to clonidine for tic suppression. The trials emphasize the importance of selecting treatments based on efficacy and side effect profiles.

**Table 2:** Data from https://clinicaltrials.gov as of February 06, 2025.

Study title	Brief summary	Condition(s)	Interventions	Age group	Study design	Location(s)
Phase 2 and 3						
Alternatives for Reducing Tics in Tourette syndrome (TS): A Study of TEV-50717 (Deutetrabenazine) for the Treatment of Tourette syndrome in Children and Adolescents	This study assesses the efficacy and safety of deutetrabenazine (TEV-50717) tablets in reducing motor and phonic tics associated with Tourette syndrome (TS) in children and adolescents aged 6 to 16 years.	Tourette syndrome	Deutetrabenazine and placebo	Children	Randomised parallel-group quadruple-blinded clinical trial for treatment.	United States, Canada, Denmark, Russian Federation, Serbia and Spain
Phase 3						
A Study to Test if TEV-50717 is Effective in Relieving Tics Associated with Tourette syndrome (TS)	A placebo-controlled, double-blind, randomized study using a 1:1:1 allocation ratio was conducted to assess the efficacy of TEV-50717 (low-dose and high-dose) in reducing tics in Tourette syndrome (TS) patients.	Tourette syndrome	Deutetrabenazine and placebo	Children	Randomised parallel-group quadruple-blinded clinical trial.	United States, Argentina, Australia, Colombia, Hungary, Italy, Korea, Mexico, Poland and Ukraine
Study Evaluating the Safety and Efficacy of Fixed-dose Once-daily Oral Aripiprazole in Children and Adolescents with Tourette Disorder	The goal of the current trial is to determine the efficacy and safety of Once-daily aripiprazole in reducing Total Tic Severity in children and adolescents with Tourette Disorder.	Tourette disorder and Tic disorder	Aripiprazole and Placebo	Children	Randomised parallel-group quadruple-blinded clinical trial	United States, Canada, Germany, Hungary, Italy, Mexico, Romania, Spain, Sweden.
Safety and Tolerability of Once-daily Oral Aripiprazole in Children and Adolescents with Tourette Disorder	The goal of the current trial is to determine the safety of Once-daily aripiprazole in reducing Total Tic Severity in children and adolescents with Tourette Disorder.	Tourette disorder and Tic disorder	Aripiprazole	Children and adults.	Non-randomized single-group open-label clinical trial.	United States, Canada, Hungary, Italy.
Efficacy & Safety Study of Once-weekly Oral Aripiprazole in Children and Adolescents with Tourette Disorder (TD)	This trial aims to evaluate the efficacy and safety of once-weekly aripiprazole in reducing Total Tic Severity (TTS) scores in children and adolescents with Tourette Disorder.	Tourette disorder	Aripiprazole and placebo	Children	Randomised parallel-group quadruple-blinded clinical trial	United States, Bulgaria, Germany, Romania and Ukraine
Efficacy & Safety Study of Once-weekly Oral Aripiprazole in Children and Adolescents with Tourette Disorder	This study aims to evaluate the efficacy and safety of once-weekly aripiprazole in reducing Total Tic Severity (TTS) scores in children and adolescents with Tourette Disorder.	Tourette Disorder	Aripiprazole and placebo	Children	Randomised parallel-group quadruple-blinded clinical trial	United States, Canada, Hungary, Korea, Mexico, and Taiwan.
Safety and Tolerability Study of Once-weekly Oral Aripiprazole in Children and Adolescents with Tourette Disorder	This trial aims to evaluate the long-term efficacy, safety, and tolerability of once-weekly aripiprazole in children and adolescents with Tourette Disorder.	Tourette Disorder	Aripiprazole	Children and adults	Non-randomized single-group open-label clinical trial	United States, Bulgaria, Canada, Germany, Hungary, Korea, Mexico, Romania, Taiwan and Ukraine
Aripiprazole in Children and Adolescents with Chronic Tic Disorder or Tourette Disorder	This trial aims to evaluate the efficacy and safety of aripiprazole in children and adolescents (ages 6–18) with chronic tic disorders or Tourette disorder.	Chronic Motor or Vocal Tic Disorder, Tourette Disorder.	Aripiprazole and placebo	Children and adults.	Randomised parallel-group quadruple-blinded clinical trial	Korea
A Randomized, Placebo-controlled, Tourette syndrome Study.	This study evaluates whether topiramate improves tics, attention, and obsessive-compulsive symptoms in Tourette syndrome based on prior reports of its effectiveness.	Tourette syndrome	Topiramate and placebo	Children, adults and older adults.	Randomised parallel-group quadruple-blinded clinical trial	United States
An Open-label Study to Determine the Efficacy and Safety of Topiramate in the Treatment of Tourette syndrome.	This study evaluates the effectiveness of topiramate in reducing motor tics and associated symptoms, such as attention deficits and obsessive-compulsive behaviors, in individuals with Tourette syndrome.	Tourette syndrome		Children, adults and older adults.	Non-randomized single-group open-label clinical trial	
**Phase 4**						
Effects of Ondansetron in Obsessive–compulsive and Tic Disorders	This randomized study evaluates 4 weeks of ondansetron (24 mg/day) vs placebo on symptoms and brain function in OCD and tic disorder patients, with MRI scans and assessments at baseline, Week 2, and Week 4. It hypothesizes more significant symptom reduction and increased brain activation with ondansetron.	Obsessive–Compulsive Disorder (OCD), Tic Disorders, and Tourette syndrome.	Ondansetron and placebo	Adults	A randomized, parallel-group, quadruple-blinded clinical trial.	United States
Guanfacine in Children with Tic Disorders	This pilot study aims to assess the preliminary tolerability and efficacy of extended-release guanfacine in children with Tourette Disorder (Tourette syndrome).	Tourette syndrome	Placebo, Extended-Release Guanfacine	Children	Randomised parallel-group quadruple-blinded clinical trial.	United States
Developing New Treatments for Tourette syndrome: Therapeutic Trials with Modulators of Glutamatergic Neurotransmission	This pilot study evaluates Riluzoe and D-serine for tic suppression in Tourette syndrome (TS), comparing 8 weeks of treatment vs. placebo. It assesses tic reduction (YGTSS) and psychiatric symptoms, with safety monitoring, including vitals, lab tests, and adverse events, informing glutamate-targeted therapies.	Tourette syndrome	D-serine, Riluzoe and Placebo	Children	Randomised parallel-group quadruple-blinded clinical trial	United States
Comparison of Keppra and Clonidine in the Treatment of Tics	This study aims to compare the tic-suppressing efficacy and safety of levetiracetam versus clonidine in a 15-week placebo run-in, double-blind, crossover trial, hypothesizing that levetiracetam will be more effective with fewer side effects than clonidine.	Tic Disorders, Tourette syndrome	Levetiracetam and Clonidine	Children and adults	Randomised crossover triple-blinded clinical trial for treatment.	United States
Open-Label Trial of Aripiprazole in Children and Adolescents with Tourette Disorder	This study aims to evaluate the efficacy of aripiprazole in reducing tics in children and adolescents (ages 7–18) with Tourette Disorder (TD) or chronic motor tic disorder.	Tourette syndrome, Tic Disorders	Aripiprazole	Children and adults	Non-randomized single-group open-label clinical trial	United States

### 3.4. Comparative analysis of clinical trials and drug efficacy

An overview of clinical trials and the efficacy of pharmacological treatments for TS is presented, comparing mechanisms of action, treatment outcomes, and adverse effects associated with these medications. Table 3 summarizes clinical trials evaluating pharmacological treatments for TS, focusing on efficacy, safety, and tolerability. Aripiprazole demonstrated the most consistent tic reduction across multiple trials, with higher doses proving more effective but increasing sedation and weight gain. Deutetrabenazine (TEV-50717) showed limited efficacy, with no significant advantage over placebo and frequent side effects such as fatigue and nausea. Topiramate exhibited tic reduction, particularly for motor tics, but was associated with cognitive slowing. Ondansetron and glutamatergic modulators (D-serine, riluzole) showed potential but require further validation. Guanfacine and clonidine were beneficial for ADHD-related symptoms, though sedation was a common concern.

**Table 3:** Comparative results of clinical trials investigating pharmacological interventions of Tourette syndrome, adopted from https://clinicaltrials.gov as of February 06, 2025.

Study title	Study parts	Dose regimens	Enrolment	Baseline characteristics	Adverse events	Conclusion
Alternatives for Reducing Tics in Tourette syndrome (TS): A Study of TEV-50717 (Deutetrabenazine) for the Treatment of Tourette syndrome in Children and Adolescents	Phase 2/3, Randomized, Double-blind	TEV-50717 (6–48 mg/day) vs. Placebo, 12 weeks	114 Completed: TEV-50717: 54 Placebo: 56 Dropouts: (4) Due to adverse events or withdrawal	Mean age: 11.5 years, 85.7% White, 48.7% Male	Higher in TEV-50717 group (fatigue, weight gain, nausea, somnolence, headache); no serious AEs	TEV-50717 showed no significant advantage over placebo for tic reduction; AEs were more frequent in the TEV-50717 group
A Study to Test if TEV-50717 is Effective in Relieving Tics Associated with Tourette syndrome (ARTISTS2)	Placebo-controlled, double-blind, fixed-dose study	Low-dose (up to 36 mg/day) & High-dose (up to 48 mg/day)	158 Completed: 145 Dropouts (13): Due to adverse events, withdrawal, or protocol violations	Mean age: 11.7 years, 75.3% male	36.54% had adverse events, most common: somnolence, headache, nausea	TEV-50717 improved tic severity but was not statistically significant vs placebo
Study Evaluating the Safety and Efficacy of Fixed-dose Once-daily Oral Aripiprazole in Children and Adolescents with Tourette Disorder	Randomised, double-blind, placebo-controlled	Low-dose (5–10 mg/day) & High-dose (10–20 mg/day)	133 Completed: 119 Dropouts (14): Due to adverse events, withdrawal, or protocol violations	Mean age: 11.5 years, 78.2% male	47.73% (low-dose) & 64.44% (high-dose) had adverse events, sedation & somnolence common	Aripiprazole significantly reduced tic severity vs. placebo, with high-dose more effective
Safety and Tolerability of Once-daily Oral Aripiprazole in Children and Adolescents with Tourette Disorder	Open-label, Single-Group Assignment	Aripiprazole once-daily, flexible dosing	110 Completed: 75 Dropouts (35): Due to adverse events, withdrawal, or other reasons	Mean age: 11.7 years, Male: 78.2%	Typical: Weight gain (23.6%), somnolence (11.8%), nausea (7.3%)	Aripiprazole was generally well tolerated; there was moderate improvement in tic severity
Efficacy & Safety Study of Once-weekly Oral Aripiprazole in Children and Adolescents with Tourette Disorder (TD)	Randomised, Double-Blind, Parallel Assignment	Aripiprazole 52.5 mg, 77.5 mg, 110 mg, and Matching Placebo (Once-Weekly, 8 weeks)	83 Completed: 68 Dropouts (15): Due to loss to follow-up, adverse events, or consent withdrawal	Mean Age: 11.9 years; 75.9% Male; Mean BMI: 20.0 kg/m²; YGTSS Total Tic Score: 30.2	common: Somnolence (11.82%), Headache (10.00%), Fatigue (10.00%), Weight Gain (23.64%)	Aripiprazole effectively reduced tic severity but with notable side effects, especially at higher doses.
Efficacy & Safety Study of Once-weekly Oral Aripiprazole in Children and Adolescents with Tourette Disorder	Pretreatment Phase, Treatment Phase, Follow-up Period	Aripiprazole 52.5 mg, 77.5 mg, 110 mg, Placebo, orally once-weekly for 8 weeks	135 Randomised: 90 to Aripiprazole, 45 to Placebo Completed: 78 (Aripiprazole), 35 (Placebo) Dropouts: 12 (Aripiprazole), 10 (Placebo)	Mean Age: 11.9 years, 77% Male; Mean Baseline YGTSS Total Tic Score: 30.1	42.2% of the Aripiprazole group reported adverse events (e.g., nausea, fatigue, headache); Serious AEs: 3.3%	Aripiprazole demonstrated significant improvement in tic severity compared to placebo. The treatment was generally well tolerated, but some participants experienced mild to moderate adverse events.
Safety and Tolerability Study of Once-weekly Oral Aripiprazole in Children and Adolescents with Tourette Disorder	Open-label, 52-week Extension Study	52.5 mg QW, 77.5 mg QW, 110 mg QW	170 participants Completed: 89 Dropouts (81): Sponsor discontinued (43), consent withdrawal (12), adverse events (6)	Mean Age: 12.2 years, 73.5% Male, 26.5% Female, Mean BMI: 21.0 kg/m²	Common: Weight Gain (8.2%), Somnolence (6.5%), Headache (11.2%). Severe: Suicidal Ideation, Diabetes.	Long-term use was generally safe; improvements in tic severity were sustained. Monitoring is required for metabolic changes.
Aripiprazole in Children and Adolescents with Chronic Tic Disorder or Tourette Disorder	Randomised, Double-Blind, Placebo-Controlled, Parallel Assignment	2–20 mg/day PO for 10 weeks	61 participants (32 Aripiprazole, 29 Placebo) Completed: 54 (29 Aripiprazole, 25 Placebo) Dropouts: 7	Mean Age: 10.95 years, Male: 86.9%, Female: 13.1%	Common: Nausea, Headache, Akathisia; Serious: None reported	Aripiprazole improved tic severity vs placebo but with some side effects
A Randomized, Placebo-controlled, Tourette syndrome Study.	Randomised, placebo-controlled	Topiramate (25–200 mg) vs. Placebo	29 participants (15 Topiramate, 14 Placebo) Completed: 20 Dropouts: 9	Mean age: 16.5 years, 89.7% male	Topiramate: 73.3% (GI issues, headache, drowsiness, kidney stones); Placebo: 57.1%	Topiramate reduced tic severity (*P* = .0259) but had mild adverse effects. Further research is needed.
An Open-label Study to Determine the Efficacy and Safety of Topiramate in the Treatment of Tourette syndrome.	Single-group open-label vs. Placebo	Topiramate (25–200 mg)	20 (all completed the trial)	Mean age: 16.5 years, 90% male	Common: Headache (15%), Cognitive slowing (5%). Severe: None reported.	Topiramate showed tic reduction (-14.29 TTS) but was limited by open-label design and small sample size. Publication not pursued.
Effects of Ondansetron in Obsessive–compulsive and Tic Disorders	Randomised, placebo-controlled	Ondansetron 24 mg/day vs. Placebo	110 participants Completed: 51 Dropouts (11): Lost to follow-up (4), adverse events (3), consent withdrawal (4)	Mean age: 30 years, 52.9% female	Common: Constipation (51.5%), Headache (9.1%), Dizziness (6.1%). Severe: None reported.	Ondansetron showed modest tic and OCD symptom improvement but with gastrointestinal side effects. Further studies required
Guanfacine in Children with Tic Disorders	Randomised, placebo-controlled	Extended-release Guanfacine (1–4 mg/day) vs. Placebo	34 (All completed the trial)	Mean age: 11.1 years, 67.6% male	Common: Fatigue (87.5%), Drowsiness (75%), Dry Mouth (62.5%), Headache (62.5%). Severe: Depressed mood (6.25%).	Guanfacine showed tic reduction but was associated with significant fatigue and sedation. Further research is needed.
Developing New Treatments for Tourette syndrome: Therapeutic Trials with Modulators of Glutamatergic Neurotransmission	Randomised, placebo-controlled	D-serine (30 mg/kg/day) vs. Riluzole (50–200 mg/day) vs. Placebo	39 participants Completed: 24 Dropouts (6): Lost interest (3), adverse events (2), other (1)	Mean age: 13.5 years, 87.5% male	Common: Mild GI discomfort and fatigue. Severe: None reported.	D-serine and Riluzole showed some tic reduction, but the small sample size limited findings. Further research is needed.
Comparison of Keppra and Clonidine in the Treatment of Tics	Randomised, crossover-controlled	Levetiracetam (10–50 mg/kg/day) vs. Clonidine (0.05–0.4 mg/day)	12 participants Completed: 10 Dropouts (2): Not randomized.	Mean age: 14.9 years, 70% male	Common: Fatigue, Irritability, Sleep disturbances. Severe: None reported.	There was no significant difference between treatments in tic reduction; clonidine was associated with more fatigue. Further studies are required.
Open-Label Trial of Aripiprazole in Children and Adolescents with Tourette Disorder	Open-label, single-group	Aripiprazole (1.25–10 mg/day)	11 (All completed the trial)	Mean age: 13.4 years, 90.9% male	Common: Headache (100%), Stomach discomfort (81.8%), Dizziness (81.8%), Fatigue (72.7%), Weight changes (63.6%). Severe: None reported.	Aripiprazole significantly reduced tic severity (*P* < .05), but the small sample size and open-label design limited the study. Further research is needed.

### 3.5. Comparison of efficacy and safety of medications

Table 4 compares clinical trials investigating various pharmacological treatments for TS, highlighting differences in efficacy, safety, and tolerability. Aripiprazole showed consistent tic reduction across multiple trials, with higher doses proving more effective but also causing increased sedation and weight gain. Deutetrabenazine (TEV-50717), despite some improvement in tic severity, did not show a significant advantage over placebo and had higher rates of fatigue and nausea. Topiramate demonstrated tic reduction with mild side effects, while ondansetron and glutamatergic modulators (D-serine, riluzole) showed potential but lacked strong evidence. Guanfacine was effective but caused significant drowsiness, and levetiracetam and clonidine had similar efficacy, with clonidine linked to more fatigue. The table underscores the trade-offs between efficacy and adverse effects, emphasizing the need for personalized treatment selection based on tolerability and clinical response.

**Table 4:** Comparison of Significance in clinical studies on Tourette syndrome treatments.

Study title	Outcome measure	Effect size	*P*-value	Statistical method
Alternatives for Reducing Tics in Tourette syndrome (TS): A Study of TEV-50717 (Deutetrabenazine) for the Treatment of Tourette syndrome in Children and Adolescents	Change in YGTSS TTS at Week 12	-0.07	.692	Mixed Models Analysis
A Study to Test if TEV-50717 is Effective in Relieving Tics Associated with Tourette syndrome (ARTISTS2)	Change in YGTSS TTS at Week 8 (High-Dose vs. Placebo)	-0.07	.600	
Study Evaluating the Safety and Efficacy of Fixed-dose Once-daily Oral Aripiprazole in Children and Adolescents with Tourette Disorder	Change in YGTSS Total Tic Score	0.74	.0020	
Safety and Tolerability of Once-daily Oral Aripiprazole in Children and Adolescents with Tourette Disorder	Change in Body Weight	0.50	<.05	Paired *t* test
Efficacy & Safety Study of Once-weekly Oral Aripiprazole in Children and Adolescents with Tourette Disorder (TD)	Change in YGTSS Total Tic Score	0.63	.0028	Mixed Effect Repeated Measure Model
		0.58	.0028	
Safety and Tolerability Study of Once-weekly Oral Aripiprazole in Children and Adolescents with Tourette Disorder	Change in YGTSS Total Tic Score	0.62	<.01	Paired *t* test
Aripiprazole in Children and Adolescents with Chronic Tic Disorder or Tourette Disorder	Mean Change in K-YGTSS TTS to Week 10	Not Reported		
A Randomized, Placebo-controlled, Tourette syndrome Study.	Total Tic Score (TTS) at Day 70	0.92	.0259	*t* test, two-sided
An Open-label Study to Determine the Efficacy and Safety of Topiramate in the Treatment of Tourette syndrome.	Change in TTS (Total Tic Score)	1.36	Not reported	
Effects of Ondansetron in Obsessive–compulsive and Tic Disorders	Brain activation, sensory phenomena, OCD, and tic severity changes.	Not reported		
Guanfacine in Children with Tic Disorders	Change in YGTSS Total Tic Score	0.14	.61	*t* test
Developing New Treatments for Tourette syndrome: Therapeutic Trials with Modulators of Glutamatergic Neurotransmission	Change in Total Tic Subscale (TTS)	0.59 (D-serine), 0.83 (Riluzole), 0.91 (Placebo)	.034	ANOVA
	Change in YGTSS Total Score	0.75 (D-serine), 0.86 (Riluzole), 0.72 (Placebo)	.041	
	Change in CGI-I Score	0.42 (D-serine), 0.68 (Riluzole), 0.59 (Placebo)	.028	
	Change in PGI-I Score	0.30 (D-serine), 0.42 (Riluzole), 0.48 (Placebo)	.045	
	Change in CY-BOCS Score	0.64 (D-serine), 0.52 (Riluzole), 0.32 (Placebo)	.021	
	Change in Plasma Glutamic Acid	0.51 (D-serine), 0.79 (Riluzole), 0.69 (Placebo)	.039	*t* test
	Change in Plasma Serine	0.88 (D-serine), 0.45 (Riluzole), 0.22 (Placebo)	.047	
	Change in DuPaul ADHD Rating Scale	0.55 (D-serine), 0.60 (Riluzole), 0.71 (Placebo)	.033	ANOVA
	Change in CDI-S Score	0.29 (D-serine), 0.31 (Riluzole), 0.54 (Placebo)	.049	
	Change in MASC Score	0.38 (D-serine), 0.61 (Riluzole), 0.41 (Placebo)	.042	Not Reported
Comparison of Keppra and Clonidine in the Treatment of Tics	Tic severity, psychiatric symptoms, side effects, and treatment tolerability	Not Reported		
Open-Label Trial of Aripiprazole in Children and Adolescents with Tourette Disorder	YGTSS Global Severity Score	1.91	.003	Wilcoxon (Mann–Whitney)
	Clinical Global Impression Severity Scores	2.45	.004	

Figure S1, Supplemental Digital Content, https://links.lww.com/MD/P123 illustrates that medications for TS are rated on a 1 to 10 scale, with higher scores representing greater efficacy. Aripiprazole (8/10 for motor and vocal tic reduction) is the most effective but has moderate side effects (5/10). Clonidine and Guanfacine (6/10 for vocal tics) help ADHD patients but cause sedation (6/10). Topiramate (6/10 for motor tics) has cognitive side effects (6/10). Deutetrabenazine (3/10 for tic reduction, 7/10 for side effects) showed no significant benefit. Levetiracetam and Ondansetron (4/10 for tic reduction, 3–4/10 for side effects) were better tolerated. D-serine and Riluzole (3–4/10) need further study. The chart highlights the efficacy versus side effect trade-off for treatment decisions.

## 4. Discussion

### 4.1. Main findings

This review of Phase III and IV clinical trials provides valuable insights into the pharmacological management of TS across children, adults, and older adults. The trials assessed medications targeting distinct neurotransmitter systems, including dopaminergic, adrenergic, serotonergic, glutamatergic, and GABAergic pathways. Aripiprazole, the most extensively studied medication, effectively reduced motor and vocal tics. Other medications, such as deutetrabenazine, levetiracetam, clonidine, guanfacine, ondansetron, topiramate, and newer compounds like D-serine and riluzole, showed varying results. While some medications demonstrated significant improvements in tic reduction, others, such as deutetrabenazine and levetiracetam, presented limited efficacy or inconsistent results. This variability underscores the complexity of managing TS and suggests that no single treatment is universally effective for all patients.

### 4.2. Drug treatment efficacy

Aripiprazole emerged as the most effective and widely studied drug in the reviewed trials. As a partial dopamine agonist, aripiprazole helps balance dopamine levels, thereby reducing both motor and vocal tics. It has a favorable side effect profile compared to other medications, making it a preferred option for many clinicians. Clonidine and guanfacine, which primarily target the adrenergic system, are effective in reducing vocal tics and are particularly useful for patients with comorbid ADHD symptoms. These drugs, however, are often associated with sedation, which limits their suitability for some patients, especially those in need of alertness for work or school. Ondansetron, a serotonin antagonist, has demonstrated some benefit in reducing tics, although its gastrointestinal side effects hinder its broader application. Topiramate, which modulates GABAergic and glutamatergic neurotransmission, has effectively reduced motor tics. However, its use is limited by cognitive side effects, such as cognitive slowing, particularly concerning children and adolescents. While showing promise for tic suppression, Levetiracetam has presented mixed results across studies, suggesting that it may only benefit specific subsets of patients or be more effective when combined with other treatments.

### 4.3. Side effect analysis

Side effects remain a critical consideration when prescribing pharmacological treatments for TS. Although aripiprazole is highly effective in reducing tics, it is associated with side effects such as sedation, weight gain, and metabolic changes, particularly at higher doses. Clonidine and guanfacine, though adequate for vocal tics and ADHD symptoms, are frequently linked to sedation, which can be problematic in active adults or those requiring high cognitive function. Despite its efficacy for motor tics, Topiramate is associated with cognitive slowing, which can significantly impact a patient’s quality of life, especially in academic or professional settings. Ondansetron’s gastrointestinal side effects, such as constipation, nausea, and dizziness, complicate its use in some patients, limiting its therapeutic value. While generally well tolerated, Levetiracetam has demonstrated inconsistent results across trials, and further studies are needed to understand its role in TS treatment better. The trade-off between efficacy and side effects in these treatments highlights the importance of a careful, individualized approach to medication management for TS.

### 4.4. Individualized treatment approaches

Given the complex and heterogeneous nature of TS, individualized treatment strategies are essential. Factors such as patient age, sex, comorbidities (e.g., ADHD or obsessive–compulsive disorder), disease duration, and even genetic predispositions can significantly influence the effectiveness and tolerability of pharmacological treatments. For instance, children and adolescents with TS may respond differently to medications than adults due to age-related differences in brain development and metabolism. Additionally, medications with sedative effects, such as clonidine and guanfacine, may be less suitable for individuals with active lifestyles, whereas those with co-occurring ADHD may benefit from these treatments. The development of pharmacogenetic tools could further enhance treatment personalization, helping clinicians predict which medications are most likely to be effective for individual patients based on their genetic profiles. Further research could improve treatment outcomes and reduce the current trial-and-error process in TS management.

### 4.5. Strengths and limitations

This review’s strength lies in its focus on Phase III and IV clinical trials, which provide critical real-world evidence on the safety and efficacy of pharmacological treatments for TS. By incorporating drugs with varying mechanisms of action, this review offers a comprehensive overview of available treatment options. However, several limitations must be considered. First, the number of studies included in the analysis was limited, with only 15 relevant Phase III and IV trials meeting the inclusion criteria. This limited sample size restricts the generalization of findings to the broader TS population. Additionally, the variety of trial designs and outcome measures across studies poses a challenge in comparing results, leading to some inconsistency in the reported efficacy of treatments. The small sample sizes in many studies, particularly for medications such as D-serine and riluzole, further reduce the generalizability of the findings.

## 5. Conclusion

In conclusion, the Phase III and IV clinical trials reviewed in this study provide valuable insights into the pharmacological treatment of TS. Aripiprazole stands out as the most effective and widely studied medication, but other treatments, including clonidine, guanfacine, ondansetron, topiramate, and levetiracetam, show varying degrees of efficacy. The results emphasize the importance of considering medications’ efficacy and side effect profiles when selecting a treatment regimen for individuals with TS. Future research should focus on increasing the number of high-quality trials, exploring genetic factors that may influence treatment outcomes, and standardizing outcome measures to provide more robust data for clinicians. Additionally, individualized treatment approaches, informed by patient characteristics and pharmacogenetic data, could significantly improve the management of this complex neurodevelopmental disorder.

## Acknowledgments

The author would like to acknowledge Deanship of Graduate Studies and Scientific Research, Taif University for funding this work.

## Author contributions

**Conceptualization:** Mohammed S. Alharthi.

**Data curation:** Mohammed S. Alharthi.

**Formal analysis:** Mohammed S. Alharthi.

**Funding acquisition:** Mohammed S. Alharthi.

**Investigation:** Mohammed S. Alharthi.

**Methodology:** Mohammed S. Alharthi.

**Project administration:** Mohammed S. Alharthi.

**Software:** Mohammed S. Alharthi.

**Writing – original draft:** Mohammed S. Alharthi.

**Writing – review & editing:** Mohammed S. Alharthi.

## Supplementary Material


