# Supplementary material for: A narrative review of Phase III and IV clinical trials for the pharmacological treatment of Tourette’s syndrome in children, adults, and older adults
Source: Medicine (Baltimore). 2025 Jun 6;104(23):e42760. doi: 10.1097/MD.0000000000042760 (PMC12151041; doi:10.1097/MD.0000000000042760)
Supplement: Supplementary file 1 [file medi-104-e42760-s001.docx]

**Supplementary Figure 1:** Efficacy and Safety Comparison of Tourette’s Syndrome Medication.
